# Hospital Management

**Published:** 1906-03-24

**Authors:** 


### HOSPITAL ADMINISTRATION.

### CONSTRUCTION AND ECONOMICS.

### HOSPITAL MANAGEMENT.

#### THE EDGAR SPEYER PRIZE COMPETITION.

Copies of the essay by Mr. Godfrey Heathcote Hamilton, secretary of the National Hospital for the Paralysed, who was awarded the prize of £100 and a silver cup offered by Mr. Edgar Speyer, and of that by Mr. Ernest William Morris, secretary of the London Hospital, which the judges decided was nearly equal in merit to the prize essay, and for which a special prize of £50 was awarded, have now been issued, and may be obtained from the publisher, Mr. George Barber, 23 Furnival Street, Holborn, E.C., price 6d., post free. We took occasion to point out when the prize was first offered that it was not possible to hope to secure an exhaustive essay which would include all the points which it was essential should be dealt with in the frankest and fullest manner, unless a guarantee was given to the writers that their names should be withheld from publication. To this opinion we adhere in the interests of the hospitals and of their economical and efficient management, and we are supported in this view by several gentlemen who have the largest experience and the best right to be heard on these questions.

The object of Mr. Edgar Speyer in offering the prize was no doubt to obtain an essay which should prove of practical value to hospital managers and to the responsible officials of the voluntary hospitals. Copies of the prize essays having been published and circulated amongst the hospitals, we have much pleasure in acceding to a request that we should open the columns of The Hospital to a symposium on the subject. We are prepared to devote the necessary space to enable the economical management of an efficient voluntary hospital to be discussed in the frankest and fullest manner by those who have a practical knowledge of the subject, who will thus be afforded an opportunity of stating their experience without reservation though impersonally. Every communication must be accompanied by the name and address of the writer, but we shall be prepared to accede to the wish of any correspondent who may desire to withhold his name on being satisfied with his *bona fides.*

---

A Meeting was held at 76 Wimpole Street on the 21st inst. to inaugurate a scheme for the foundation of another Pay Hospital for gentlewomen. A committee has been formed, with Sir W. Lethbridge as chairman, and they will endeavour to raise a sum of £20,000 in order to provide the necessary initial funds.

